# Digestibility of Amino Acids in Protein-Rich Feed Ingredients Originating from Animals, Peanut Flour, and Full-Fat Soybeans Fed to Pigs

**DOI:** 10.3390/ani10112062

**Published:** 2020-11-07

**Authors:** Ayodeji Simeon Aderibigbe, Chan Sol Park, Adekunle Adebiyi, Oluyinka Abiona Olukosi, Olayiwola Adeola

**Affiliations:** 1 Department of Animal Sciences, Purdue University, West Lafayette, IN 47907, USA; aaderibi@purdue.edu (A.S.A.); park765@purdue.edu (C.S.P.); Adekunle.adebiyi@forfarmers.eu (A.A.); 2 Department of Poultry Science, University of Georgia, Athens, GA 30602, USA; oaolukosi@uga.edu

**Keywords:** amino acids, alternative feedstuff, growing pigs, ingredients, protein

## Abstract

**Simple Summary:**

Dietary protein is a major contributing factor to animal feed cost. Recently, the overwhelming demand for and the unstable cost of conventional feed ingredients, such as soybean meal, have caused interest in alternative feed ingredients. By-products of agricultural industries may be cost-effective alternatives to conventional protein sources in swine diets. Therefore, to assess their full nutritional potential for pigs, it becomes necessary to evaluate the protein quality in alternative feed ingredients. In the present work, the digestibility of amino acids in four animal-derived protein sources (egg albumen, casein, blood meal, and blood plasma meal) and two plant-derived protein sources (peanut flour and full-fat soybean) were evaluated. Experimental diets were prepared to contain each of the test ingredients as the sole source of nitrogen and fed to growing pigs in two digestibility trials. The results of the experiments showed greater digestibility of amino acids in casein than the other animal protein sources. Digestibility of most amino acids in peanut flour was greater than in full-fat soybeans. Based on the current results, it is concluded that the test ingredients contain readily digestible amino acids which could make them useful in the diets of growing pigs.

**Abstract:**

Standardized ileal digestibility (SID) of amino acids (AA) in alternative protein sources for growing pigs was determined in this study. Diets containing egg albumen (EA), casein, blood meal (BM), and blood plasma meal (BPM) and a nitrogen-free diet (NFD) were fed to 20 barrows in a quadruplicate 5 × 2 incomplete Latin square design with two periods in experiment 1. The SID of AA was greater in casein than other ingredients (*p* < 0.05), except Pro. The SID of Arg, Ile, and Met was lower (*p* < 0.05) in EA than BM and BPM. The SID of Trp in BM was greater (*p* < 0.05) than EA but not different from BPM. In experiment 2, 20 pigs were fed diets containing peanut flour (PF) or full-fat soybeans (FFSB) or NFD in a randomized complete block design with body weight as a blocking factor but providing six observations for NFD. The SID of Arg, Ileu, Leu, Met, Phe, and Val was greater (*p* < 0.05) in PF than FFSB. The SID of Lys was greater (*p* < 0.05) in FFSB than PF. In summary, the test ingredients contain readily digestible AA and could serve as alternative protein sources for growing pigs.

## 1. Introduction

The increasing demand for soybean meal (SBM) in swine nutrition, primarily due to its superior amino acid (AA) profile [1,2], is gradually shifting the radar to alternative sources of dietary protein. Many alternative feed ingredients that may be cost effective and useful in swine diets are produced by using by-products of industries involved in grain processing, oil refining, or dairy and poultry processing. Common animal-derived protein sources that have been used in swine diets are blood meal (BM), blood plasma meal (BPM), and casein, which have been shown to be highly digestible when fed to pigs [3,4]. Another potential protein supplement is egg albumen (EA), which has an excellent amino acid profile with a relatively high concentration of methionine compared to porcine plasma [5,6]. However, it has not been well investigated in swine nutrition. Other potential dietary protein sources may be of plant origin, such as peanut flour (PF) and full-fat soybean (FFSB). Although FFSB contains high concentrations of crude protein (CP) and energy [7,8], it also contains a considerable amount of trypsin inhibitors that limits its use in swine diets. Alternatively, PF produced from dehulling and grinding peanuts contains high CP [9], which makes it another potentially valuable protein source for pigs. However, there is a paucity of information on the amino acid digestibility of these aforementioned ingredients for pigs. Therefore, the objectives of this study were to determine the apparent ileal digestibility (AID) and standardized ileal digestibility (SID) of AA in EA, casein, BM, and BPM in experiment 1 (Exp. 1) and PF and FFSB in experiment 2 (Exp. 2) for growing pigs.

## 2. Materials and Methods

All experimental protocols were reviewed and approved under protocol number 1111000248 by the Purdue Animal Care and Use Committee.

### 2.1. Ingredients and Dietary Treatments

Experimental diets in both Exp. 1 and Exp. 2 were formulated to contain 190 g/kg CP, with the test ingredient supplying all of the dietary N (Table 1). Cornstarch and dextrose were added as energy sources, and additional soybean oil was added to the diet containing PF. In both experiments, a nitrogen-free diet (NFD) was fed to pigs to determine basal ileal endogenous losses (BEL) of AA. Diets were formulated to meet the vitamin and mineral requirements of pigs in the national research council (NRC) [10]. Experiment 1 consisted of diets containing EA, casein, BM, and BPM. For Exp. 2, PF and FFSB were used to prepare experimental diets. Chromic oxide was incorporated into all diets at 5 g/kg of diet as an indigestible marker.

### 2.2. Experimental Animals, Feeding and Sample Collection

Twenty crossbred barrows with an initial body weight (BW) of 20 ± 0.28 kg (Exp. 1) and 22.7 ± 1.49 kg (Exp. 2) were used for each experiment. Prior to the experiments, pigs were surgically fitted with T-cannulas at the distal ileum as previously described [11]. Subsequently, pigs were housed individually in floor pens with ad libitum access to water and 12 h of artificial lighting in climate-controlled rooms (22 °C). In Exp. 1, pigs were divided into 4 groups by initial BW. Then, pigs were fed experimental diets in a quadruplicate 5 × 2 incomplete Latin square design with 2 periods. There were 8 replicates per experimental diet. In Exp. 2, pigs were allotted to 3 experimental diets in a randomized complete block design with BW as a blocking factor, which provided 7 replicates for experimental diets containing PF and FFSB and 6 replicates for NFD. In both Exp. 1 and Exp. 2, there were 5 d of adaptation to the experimental diets, followed by a 2-d collection period of ileal digesta by attaching plastic tubular bags (Whirl-Pak bag; NASCO, Fort Atkinson, WI, USA) to the externalized T-cannulas. To reduce proliferation of bacteria in the ileal digesta samples, each bag contained 10 mL of 5% formic acid, and ileal contents were immediately stored at −20 °C between collections. After each experimental period, the ileal digesta was slightly thawed and pooled within pig and diet, subsampled, and lyophilized. Daily feed allowance was given at 4% of BW of the smallest pig in each group or block at the beginning of each period, and feed was given in 2 equal portions at 0600 and 1800.

### 2.3. Chemical Analysis

Experimental diets, test ingredients, and freeze-dried ileal digesta samples were ground to pass through a 0.5-mm screen using a centrifugal grinder before further analyses. Samples of experimental diets, test ingredients, and ileal digesta were analyzed for dry matter (DM) by drying at 105 °C for 24 h in a forced-air oven (Precision Scientific Co., Chicago, IL, USA; method 934.01) [12]. The concentration of N in experimental diets, test ingredients, and ileal digesta was analyzed by a combustion method (TruMac N; LECO Corp., St. Joseph, MI, USA; method 990.03) [13], and the concentration CP was calculated as the product of N concentration and 6.25. Gross energy in the test ingredients was analyzed using an isoperibol bomb calorimeter (model 6200; Parr Instrument Co., Moline, IL, USA). Amino acid analyses were conducted at University of Missouri Agricultural Experiment Station Chemical Laboratories (Columbia, MO, USA). Briefly, ground samples were hydrolyzed by 6 M HCl (or BaOH for Trp analysis) at 110 °C for 24 h under N atmosphere. For the sulfur-containing amino acids, performic acid oxidation was conducted prior to acid hydrolysis. The concentration of AA in samples was then analyzed by high-performance liquid chromatography after post-column derivatization (method 982.30 E (a, b, c)) [12].

The concentration of Cr in ground experimental diets and ileal digesta samples was measured by a spectrophotometry (SpectraCount, model AS1000, Packard, Meriden, CT, USA) at 450 nm of absorption after wet digestion in nitric acid and 70% perchloric acid [14]. Concentration of P in the samples was determined using a colorimetric assay after a wet-ash digestion (method 935.13) [12]. Briefly, acid molybdate and Fiske–Subbarow reducer solutions were added to the wet-ashed samples to measure the concentrations of P through the formation of a phosphomolybdenum complex. Concentration of P was determined by spectrophotometry at a wavelength of 620 nm. The Ca concentrations in the digested samples were determined by an atomic absorption spectrophotometer (AAnalyst 300; Perkin Elmer, Norwalk, CT, USA) [12].

### 2.4. Calculations and Statistical Analysis

The AID and SID of CP and AA in each test ingredient, and the BEL of AA were calculated as previously described [15]. Data were analyzed using the MIXED procedure of SAS (SAS Inst. Inc., Cary, NC, USA). In Exp. 1, the model included diet as a fixed variable and group, period, and pig within group as random variables. The model for Exp. 2 included diet as a fixed variable and block as a random variable. Least squares means were calculated using the LSMEANS statement. In Exp. 1, least squares means were separated using the PDIFF option with Tukey’s adjustment. In all statistical analyses, pig served as the experimental unit and the significance was declared at an α value of 0.05.

## 3. Results

### 3.1. Chemical Analysis

The analyzed concentration of CP ranged from 784 g/kg in BPM to 914 g/kg in BM (Table 2). The analyzed concentrations of CP and AA in experimental diets were close to the values calculated with the analyzed CP and AA in test ingredients (Table 3).

### 3.2. Basal Ileal Endogenous Losses of AA

The BEL profile for both indispensable and dispensable AA was similar for both experiments (Table 4). The BEL of indispensable AA ranged from 124 mg/kg DMI for Met to 877 mg/kg DMI for Leu (Exp. 1), and from 125 mg/kg DMI for Met to 809 mg/kg DMI for Leu (Exp. 2). The BEL of dispensable AA ranged from 272 mg/kg DMI for Cys to 3856 mg/kg DMI for Pro (Exp. 1), and from 238 mg/kg DMI for Cys to 1630 mg/kg DMI for Pro (Exp. 2).

### 3.3. Digestibility of CP and AA in Exp. 1

The AID and SID of CP and all AA were greater (*p* < 0.05) in casein than the other test ingredients, except Thr and Pro (Table 5 and Table 6). The AID and SID of Arg in EA were lower (*p* < 0.05) than in the other ingredients. The AID and SID of Arg in BPM were greater (*p* < 0.05) than in BM. The AID and SID of His in BM were lower (*p* < 0.05) than in EA but not different from BPM. The AID and SID of Lys in BPM were lower (*p* < 0.05) than in the other ingredients. The AID and SID of Ile, Met, and Glu in BM and BPM were greater (*p* < 0.05) than in EA; however, the AID and SID of Thr in EA were greater (*p* < 0.05) than in BM and BPM. The AID and SID of Trp in BM were greater (*p* < 0.05) than in EA but not different from BPM. The AID and SID of Cys and Gly in BM were lower (*p* < 0.05) than in the other ingredients.

### 3.4. Digestibility of CP and AA in Exp. 2

The AID and SID of CP, Thr, Trp, Cys, Glu, Pro, and Ser were similar for PF and FFSB (Table 7 and Table 8). Furthermore, the SID results of His in PF and FFSB were not different from each other. The AID and SID of Arg, Leu, Met, Phe, Val, Ala, and Tyr were greater (*p* < 0.05) in PF than in FFSB. The AID and SID of Lys, Asp, and Gly were greater (*p* < 0.05) in FFSB than in PF. Although the AID of Ile was similar between PF and FFSB, the SID of Ile in PF was greater (*p* < 0.05) than in FFSB.

## 4. Discussion

The demand and increasing cost of conventional nutrient-rich feed ingredients such as SBM for pigs offer a strong economic incentive to identify cost-effective alternative feed ingredients. In the current study, we tested six protein-rich feed ingredients that could serve as potential alternatives to conventional sources of dietary protein in swine diets. Overall, the test ingredients had similar nutrient composition to the values reported in the NRC [10], except for EA which has not been characterized in the NRC [10]. The concentrations of CP and AA in EA used in the current study were within the range of values previously reported [16,17], although branched-chain AA (Ile, Leu, and Val), Asp, and Glu concentrations were relatively higher than previously reported values. However, it is pertinent to state that in contrast to the current study, previous studies [16,17] used spray dried egg by-products, not exclusively from EA, which may have caused the variations observed among studies. Casein was analyzed to contain similar or slightly greater gross energy and nutrients than as provided in NRC [10], but the analyzed gross energy and nutrient concentrations were close to the values reported in another study [18]. The concentrations of gross energy and nutrients in BM and BPM were similar to the NRC [10]. However, the CP and majority of AA in the BM were greater than the values reported in the NRC [10], but were similar to another previous report [19]. The concentrations of CP and AA in FFSB were within the range of previously reported values [9,10,15]. Similar with EA, there is a paucity of data on PF, but the analyzed CP and AA concentrations agree with previous studies [9,15], except for Arg and Lys.

The BEL of CP and AA are similar between Exp. 1 and Exp. 2, but are relatively greater than previous reports [10,15,20]. The reason for the high BEL estimates was not immediately clear. One explanation is that estimates of BEL can be variable due to differences in experimental conditions among studies, such as pig genotype, intestinal health of the pig, method of digesta sampling, and analytical procedures [21,22]. Another potential reason might be the inconsistencies in the proportion of cornstarch and dextrose in the NFD used in the determination of BEL of AA in these studies. However, a previous investigation showed that the ileal AA flow in pigs was independent of cornstarch to dextrose ratios [23]. Although relatively shorter, a 5-day adaptation period was used in the current study; however, this is widely similar to other previous reports [2,3,15], and therefore, the differences observed are unlikely to be related to the length of the adaptation period to the diets. Nevertheless, the BEL values observed in the current study were still within the range of values of a summary of previous studies [24]. Furthermore, the BEL of Glu, Gly, and Pro were relatively high compared to other AA, which is similar to previous reports [2,11]. An increase in endogenous losses will result in increased SID estimates, sometimes above 100%, as was observed in the current study for Gly and Pro in Exp. 1.

Animal protein sources have served as protein supplements with a high quality of protein and AA composition, especially for young pigs. As observed in Exp. 1, EA has similar AA digestibility as BM and BPM but less AA digestibility than casein. This observation is similar to a previous report [17]. Although EA has a higher concentration of Met similar to previous reports [5,6,17], the digestibility of Met was lower compared to BM and BPM. Egg proteins have been reported to contain trypsin inhibiting ovomucoid [25,26], which can limit protein utilization, although this was not determined in the current study. We also investigated the digestibility of AA in casein, which is produced from pasteurized skim milk and is highly digestible [3,27]. As observed in this study, the SID of AA in casein agrees with previous reports [3,10,18] and was greater than the AA digestibility of EA, BM, or BPM.

Blood meal and BPM are by-products from meat production industries that may be used in the diets of pigs because of the high nutritional qualities of blood protein [28]. In the current study, BM was shown to have an average SID of AA of 80%, which is similar to previous research [19,29]. However, the values were less than the AA digestibility provided in the NRC [10], which lists the average SID of AA in BM at 88%. It is possible that the processing of BM impacts the digestibility of AA in the product. Although we are not aware of the specific drying times of the BM used in the current study, overheating during processing may be deleterious to the AA digestibility [30]. The SID of most AA in BPM was not different from the values in EA in the current study, which agrees with previous reports [10,17]. With the exception of Arg, Lys, Cys, Gly, and Tyr, the SID of AA between BM and BPM was similar. The SID of Lys in BPM was lower than in BM and other animal protein by-products in the current study. The processing of feed ingredients involving heat may cause Maillard reactions, which is the reaction between the amino group of Lys and the carbonyl group of a reducing sugar [31], thereby rendering the Lys unavailable to the pigs, and thus, the digestibility of Lys is reduced [2]. In contrast, a previous report [29] showed that BPM has more digestible AA than BM, including Lys, in diets fed to pigs. Among animal protein concentrates, it appears that the processing condition has a marked impact on AA digestibility, and may explain some of the variability among studies.

The SID of most AA was greater in PF than in FFSB, while the SID of Asp and Gly was greater in FFSB than in PF. This observation is similar to a previous report [15]. The digestibility of Lys was lower in PF than FFSB and was similar to a previous report [15], while the reason for this observation remains unclear. One explanation may be the inhibitory effect of Arg on Lys digestibility, which is caused by the dependency on the same transport system in the small intestine [32]. Moreover, it was observed that the concentration of Arg in PF is five times that of Lys, compared to approximately 1:1 in FFSB, which gives more credence to the possibility of AA antagonism between Arg and Lys in PF. The digestibility of AA in FFSB was similar to values in previous reports [10,15], which may indicate that the potential negative effects of residual trypsin inhibitor activity (TIA) in FFSB was negligible in the current study, although it was not analyzed in the current study. Previous reports have shown that residual TIA in heat-treated FFSB did not affect the SID of CP or AA compared to conventional SBM, despite greater values for TIA in FFSB [33]. However, substantial variations in AA digestibility in FFSB have been observed in previous studies [8,34] and these discrepancies among studies could be attributed to differences in processing or variety of FFSB or both.

## 5. Conclusions

The AID and SID of AA in casein were greater than in other animal protein sources used in the current study. Furthermore, the AID and SID of the majority of the AA in egg albumen, blood meal, and blood plasma meal were similar. The AID and SID of the majority of the AA in peanut flour were relatively greater than in full-fat soybeans. However, similar to blood plasma meal, the digestibility of Lys in peanut flour was relatively lower than in full-fat soybeans, and this warrants further investigations. The current study shows that the test ingredients contain readily digestible AA for pigs and indicates their potential as alternative protein sources for pigs.

## Figures and Tables

**Table 1:** Ingredient composition of experimental diets, g/kg as-fed basis ^1^.

	Experiment 1					Experiment 2		
Ingredient	EA	Casein	BM	BPM	NFD	PF	FFSB	NFD
Cornstarch	604.0	626.0	621.0	599.0	748.0	536.0	446.0	769.0
Egg albumen	233.0	-	-	-	-	-	-	-
Casein	-	212.0	-	-	-	-	-	-
Blood meal	-	-	213.0	-	-	-	-	-
Blood plasma meal	-	-	-	243.0	-	-	-	-
Peanut flour	-	-	-	-	-	380.0	-	-
Full-fat soybeans	-	-	-	-	-	-	495.0	-
Dextrose	100.0	100.0	100.0	100.0	100.0	-	-	100.0
Soybean oil	-	-	-	-	30.0	25.0	-	30.0
Monocalcium phosphate	25.0	22.0	26.0	14.0	29.0	15.0	15.0	20.0
Limestone	6.0	8.0	7.0	11.8	6.0	8.0	8.0	5.0
Cellulose ^2^	-	-	-	-	50.0	-	-	40.0
Salt	4.0	4.0	4.0	4.0	4.0	3.0	3.0	3.0
Potassium carbonate	-	-	-	-	4.0	4.0	4.0	4.0
Magnesium oxide	-	-	-	-	1.0	1.0	1.0	1.0
Vitamin premix ^3^	2.0	2.0	2.0	2.0	2.0	2.0	2.0	2.0
Mineral premix ^4^	1.0	1.0	1.0	1.0	1.0	1.0	1.0	1.0
Selenium premix ^5^	1.0	1.0	1.0	1.0	1.0	1.0	1.0	1.0
Chromic oxide premix ^6^	25.0	25.0	25.0	25.0	25.0	25.0	25.0	25.0

^1^ EA—Egg albumen; BM—blood meal; BPM—blood plasma meal; NFD—nitrogen-free diet; PF—peanut flour; FFSB—full-fat soybean. ^2^ Solka-Floc 40 FCC (International Fiber Corporation, Urbana, OH, USA). ^3^ Provided the following quantities per kilogram of diet: vitamin A—2640 IU; vitamin D_3_—264 IU; vitamin E—17.6 IU; vitamin K activity—2.4 mg; menadione—880 μg; vitamin B_12_—15.4 μg; riboflavin—3.52 mg; D-pantothenic acid—8.8 mg; niacin—13.2 mg. ^4^ Provided the following quantities per kilogram of diet: Cu, 9 mg as copper chloride; I, 0.36 mg as ethylenediamine dihydroiodide; Fe, 194 mg as ferrous carbonate; Mn, 17 mg as manganese oxide; and Zn, 149 mg as zinc oxide. ^5^ Supplied 300 μg of Se per kilogram of diet. ^6^ Supplied 5 g chromic oxide plus 20 g cornstarch.

**Table 2:** Analyzed nutrient composition of test ingredients, g/kg as-fed basis ^1^.

	Experiment 1				Experiment 2	
Item	EA	Casein	BM	BPM	PF	FFSB
Dry matter	949.0	913.0	919.0	938.0	953.0	939.0
Gross energy, MJ/kg	26.3	23.8	22.2	19.9	-	-
Crude protein (N × 6.25)	870.0	894.0	914.0	784.0	503.0	386.0
Ca	2.8	2.1	0.6	1.4	-	-
P	6.8	6.8	2.2	12.6	-	-
Indispensable amino acid						
Arg	49.5	27.8	44.8	46.9	51.6	29.0
His	21.3	26.7	63.7	25.6	10.4	10.1
Ile	45.2	51.1	12.5	24.5	15.4	18.2
Leu	72.3	87.8	114.2	75.6	30.7	30.1
Lys	59.8	71.9	78.1	73.3	10.6	24.9
Met	31.0	28.3	7.6	9.5	5.0	5.4
Phe	52.4	46.4	57.9	42.6	5.0	5.4
Thr	38.2	40.8	33.7	52.1	11.7	14.4
Trp	14.3	12.8	11.8	16.1	5.0	5.5
Val	59.4	61.1	75.5	54.8	19.1	19.6
Dispensable amino acid						
Ala	53.1	27.4	67.9	41.7	18.1	16.4
Asp	90.0	62.6	102.0	81.0	53.7	43.6
Cys	22.3	3.4	12.8	24.3	5.1	5.4
Glu	111.6	201.4	90.2	108.9	86.3	66.0
Gly	30.3	16.8	43.9	27.9	26.6	16.5
Pro	30.5	108.5	39.8	41.9	19.2	19.2
Ser	50.0	42.7	40.6	45.7	20.2	16.8
Tyr	32.7	49.5	27.0	40.1	18.4	13.9

^1^ EA—egg albumen; BM—blood meal; BPM—blood plasma meal; PF—peanut flour; FFSB—full-fat soybean.

**Table 3:** Analyzed nutrient composition of experimental diets, g/kg as-fed basis ^1^.

	Experiment 1					Experiment 2		
Item	EA	Casein	BM	BPM	NFD	PF	FFSB	NFD
Crude protein (N × 6.25)	217.0	191.0	206.0	193.0	30.0	194.0	183.0	10.0
Ca	6.8	7.2	6.7	6.9	7.1	6.9	7.0	5.1
P	6.1	6.1	5.8	6.3	6.1	5.4	5.8	4.2
Indispensable amino acid								
Arg	10.3	15.0	14.5	20.8	0.2	19.3	13.0	0.1
His	5.2	6.0	5.5	5.0	4.0	3.9	4.8	0.1
Ile	11.7	9.7	9.6	9.4	0.2	5.8	8.3	0.1
Leu	22.4	16.6	16.2	13.5	0.4	11.8	14.0	0.4
Lys	17.2	13.6	12.7	8.8	0.2	4.0	11.7	0.1
Met	4.7	2.8	2.4	3.7	0.1	1.9	2.5	0.2
Phe	14.0	11.0	11.0	10.6	0.3	9.2	9.1	0.1
Thr	12.5	8.3	7.5	8.0	0.1	4.5	6.6	0.2
Trp	2.7	2.3	2.8	3.0	0.4	2.0	2.5	0.4
Val	13.9	10.2	9.8	11.2	0.2	7.2	9.0	0.2
Dispensable amino acid								
Ala	10.9	9.5	8.5	10.5	0.3	7.0	7.8	0.2
Asp	26.5	24.1	23.8	21.0	0.3	20.7	19.8	0.2
Cys	2.8	2.8	2.4	3.7	0.1	1.9	2.4	0.1
Glu	23.1	38.8	38.9	44.6	0.5	34.0	30.9	0.6
Gly	10.6	9.1	8.2	13.1	0.2	10.2	7.8	0.1
Pro	10.3	10.8	10.4	8.2	0.2	7.3	8.2	0.2
Ser	10.2	9.6	9.4	9.2	0.2	8.0	7.5	0.1
Tyr	10.5	6.7	6.2	5.3	0.2	6.4	6.3	0.1

^1^ EA—Egg albumen; BM—blood meal; BPM—blood plasma meal; NFD—nitrogen-free diet; PF—peanut flour; FFSB—full-fat soybean.

**Table 4:** Basal ileal endogenous losses of crude protein and amino acids (mg/kg of dry matter intake (DMI)) at the terminal ileum of pigs fed a nitrogen-free diet.

	Experiment 1 ^1^		Experiment 2 ^2^	
Item	Mean	SD ^3^	Mean	SD
Crude protein, g/kg DMI	19.2	3.78	18.9	6.09
Indispensable amino acid				
Arg	723	88.7	630	256.5
His	313	66.5	257	91.3
Ile	464	129.2	449	179.4
Leu	877	219.4	809	310.6
Lys	774	188.7	729	330.4
Met	124	54.6	125	51.7
Phe	540	139.9	461	176.4
Thr	820	199.9	697	256.8
Trp	195	63.7	176	52.2
Val	664	176.1	625	224.9
Dispensable amino acid				
Ala	743	128.7	686	258.1
Asp	1130	268.5	1119	417.1
Cys	272	66.1	238	82.6
Glu	1332	317.8	1331	494.1
Gly	1474	302.7	1427	438.2
Pro	3856	1362.9	1630	245.3
Ser	754	195.9	638	232.5
Tyr	371	92.9	359	172.5

^1^ Each least squares mean represents eight observations. ^2^ Each least squares mean represents six observations. ^3^ SD—standard deviation.

**Table 5:** Apparent ileal digestibility (%) of crude protein and amino acids for experiment 1 ^1,2^.

Item	EA	Casein	BM	BPM	SEM	*p*-Value
Crude protein	79.4 ^b^	91.5 ^a^	73.9 ^b^	76.3 ^b^	1.66	<0.001
Indispensable amino acid						
Arg	75.9 ^d^	96.2 ^a^	82.9 ^c^	91.3 ^b^	0.92	<0.001
His	78.2 ^bc^	94.7 ^a^	67.3 ^d^	77.4 ^cd^	2.72	<0.001
Ile	79.1 ^c^	93.9 ^a^	87.8 ^b^	85.1 ^b^	1.16	<0.001
Leu	83.3 ^b^	94.7 ^a^	75.8 ^b^	75.6 ^b^	2.42	<0.001
Lys	82.2 ^b^	93.6 ^a^	78.6 ^b^	61.2 ^c^	2.65	<0.001
Met	66.9 ^c^	94.2 ^a^	85.9 ^b^	87.8 ^b^	1.24	<0.001
Phe	80.9 ^b^	95.4 ^a^	82.6 ^b^	82.5 ^b^	1.72	<0.001
Thr	83.1 ^a^	88.4 ^a^	68.4 ^b^	62.9 ^b^	2.41	<0.001
Trp	72.6 ^c^	92.0 ^a^	83.1 ^b^	77.2 ^bc^	1.68	<0.001
Val	78.1 ^b^	92.7 ^a^	70.2 ^b^	76.9 ^b^	2.35	<0.001
Dispensable amino acid						
Ala	76.0 ^bc^	92.3 ^a^	71.1 ^c^	79.6 ^b^	1.95	<0.001
Asp	82.1 ^b^	94.8 ^a^	79.8 ^b^	79.6 ^b^	1.45	<0.001
Cys	56.8 ^bc^	90.6 ^a^	21.2 ^d^	66.9 ^b^	2.53	<0.001
Glu	75.0 ^c^	94.9 ^a^	87.6 ^b^	87.7 ^b^	0.95	<0.001
Gly	79.7 ^b^	88.6 ^a^	63.3 ^c^	78.8 ^b^	1.96	<0.001
Pro	75.5	84.1	62.2	65.2	6.26	0.040
Ser	71.3 ^b^	90.7 ^a^	69.3 ^b^	69.4 ^b^	2.11	<0.001
Tyr	83.8 ^b^	93.7 ^a^	80.2 ^b^	69.2 ^c^	2.39	<0.001

^a–d^ Within a row, means without a common superscript differ (*p* < 0.05). ^1^ Each least squares mean represents eight observations. ^2^ EA—egg albumen; BM—blood meal; BPM—blood plasma meal; SEM—standard error of the mean.

**Table 6:** Standardized ileal digestibility (%) of crude protein and amino acids for experiment 1 ^1,2^.

Item	EA	Casein	BM	BPM	SEM	*p*-Value
Crude protein	87.6 ^b^	100.7 ^a^	82.5 ^b^	85.5 ^b^	1.66	<0.001
Indispensable amino acid						
Arg	82.5 ^d^	100.6 ^a^	87.5 ^c^	94.5 ^b^	0.92	<0.001
His	83.8 ^b^	99.5 ^a^	72.6 ^c^	83.2 ^b,c^	2.72	<0.001
Ile	82.8 ^c^	98.3 ^a^	92.3 ^b^	89.7 ^b^	1.16	<0.001
Leu	86.9 ^b^	99.5 ^a^	80.8 ^b^	81.7 ^b^	2.42	<0.001
Lys	86.5 ^b^	98.9 ^a^	84.3 ^b^	69.3 ^c^	2.65	<0.001
Met	69.4 ^c^	98.3 ^a^	90.8 ^b^	90.9 ^b^	1.24	<0.001
Phe	84.5 ^b^	98.9 ^a^	87.1 ^b^	87.2 ^b^	1.72	<0.001
Thr	89.2 ^a^	97.5 ^a^	78.5 ^b^	72.4 ^b^	2.41	<0.001
Trp	79.3 ^c^	99.7 ^a^	89.5 ^b^	83.2 ^b,c^	1.68	<0.001
Val	82.6 ^b^	98.7 ^a^	76.5 ^b^	82.5 ^b^	2.35	<0.001
Dispensable amino acid						
Ala	82.4 ^b^	99.5 ^a^	79.2 ^b^	86.0 ^b^	1.95	<0.001
Asp	86.1 ^b^	99.1 ^a^	84.2 ^b^	84.6 ^b^	1.45	<0.001
Cys	65.8 ^b^	99.5 ^a^	31.8 ^c^	73.8 ^b^	2.53	<0.001
Glu	80.4 ^c^	98.1 ^a^	90.7 ^b^	90.5 ^b^	0.95	<0.001
Gly	92.7 ^b^	103.6 ^a^	79.9 ^c^	89.2 ^b^	1.96	<0.001
Pro	110.5	116.9	96.5	108.8	6.26	0.076
Ser	78.2 ^b^	97.9 ^a^	76.8 ^b^	76.9 ^b^	2.11	<0.001
Tyr	87.1 ^b^	98.8 ^a^	85.7 ^b^	75.7 ^c^	2.39	<0.001

^a–d^ Within a row, means without a common superscript differ (*p* < 0.05). ^1^ Each least squares mean represents eight observations. ^2^ EA—egg albumen; BM—blood meal; BPM—blood plasma meal; SEM—standard error of the mean.

**Table 7:** Apparent ileal digestibility (%) of crude protein and amino acids for experiment 2 ^1,2^.

Item	PF	FFSB	SEM	*p*-Value
Crude protein	73.3	72.6	2.84	0.774
Indispensable amino acid				
Arg	90.5	86.1	1.43	0.006
His	77.6	81.3	2.24	0.029
Ile	79.5	78.2	2.24	0.253
Leu	84.4	77.3	2.01	<0.001
Lys	41.9	79.8	5.16	<0.001
Met	84.2	80.9	1.96	0.037
Phe	87.9	79.9	1.77	<0.001
Thr	63.0	66.4	3.68	0.203
Trp	75.5	74.6	2.61	0.457
Val	79.0	74.3	2.48	0.002
Dispensable amino acid				
Ala	77.6	70.8	2.67	0.006
Asp	73.2	77.8	2.68	0.032
Cys	61.5	62.1	3.98	0.760
Glu	83.9	84.5	1.85	0.702
Gly	48.8	60.4	4.88	0.019
Pro	73.7	70.1	5.84	0.670
Ser	71.9	72.0	2.96	0.979
Tyr	84.3	77.1	2.02	<0.001

^1^ Each least squares mean represents seven observations. ^2^ PF—peanut flour; FFSB—full-fat soybeans; SEM—standard error of the mean.

**Table 8:** Standardized ileal digestibility (%) of crude protein and amino acids for experiment 2 ^1,2^.

Item	PF	FFSB	SEM	*p*-Value
Crude protein	82.3	82.2	2.84	0.946
Indispensable amino acid				
Arg	93.5	90.6	1.43	0.034
His	83.7	86.2	2.24	0.099
Ile	86.7	83.2	2.24	0.013
Leu	90.7	82.7	2.01	<0.001
Lys	58.7	85.5	5.16	0.003
Met	90.3	85.5	1.96	0.008
Phe	92.5	84.6	1.77	<0.001
Thr	77.3	76.2	3.68	0.654
Trp	83.7	81.1	2.61	0.067
Val	87.0	80.7	2.48	<0.001
Dispensable amino acid				
Ala	86.6	78.9	2.67	0.003
Asp	78.2	83.0	2.68	0.027
Cys	73.1	71.2	3.98	0.416
Glu	87.5	87.4	1.85	0.931
Gly	61.8	77.2	4.88	0.005
Pro	94.2	88.4	5.84	0.481
Ser	79.4	79.9	2.96	0.826
Tyr	89.5	82.4	2.02	<0.001

^1^ Each least squares mean represents seven observations. ^2^ PF—peanut flour; FFSB—full-fat soybeans; SEM—standard error of the mean.
